# Reduced Graphene Oxide Decorated Na_3_V_2_(PO_4_)_3_ Microspheres as Cathode Material With Advanced Sodium Storage Performance

**DOI:** 10.3389/fchem.2018.00174

**Published:** 2018-05-23

**Authors:** Hezhang Chen, Yingde Huang, Gaoqiang Mao, Hui Tong, Wanjing Yu, Junchao Zheng, Zhiying Ding

**Affiliations:** ^1^School of Metallurgy and Environment, Central South University, Changsha, China; ^2^School of Chemistry and Chemical Engineering, Central South University, Changsha, China

**Keywords:** Na_3_V_2_(PO_4_)_3_, microspheres, reduced graphene oxide, amorphous carbon, sodium ion batteries

## Abstract

Reduced graphene oxide (rGO) sheet decorated Na_3_V_2_(PO_4_)_3_ (NVP) microspheres were successfully synthesized by spray-drying method. The NVP microspheres were embedded by rGO sheets, and the surface of the particles were coated by rGO sheets and amorphous carbon. Thus, the carbon conductive network consisted of rGO sheets and amorphous carbon generated in the cathode material. NVP microspheres decorated with different content of rGO (about 0, 4, 8, and 12 wt%) were investigated in this study. The electrochemical performance of NVP exhibited a significant enhancement after rGO introduction. The electrode containing about 8 wt% rGO (NVP/G8) showed the best rate and cycle performance. NVP/G8 electrode exhibited the discharge capacity of 64.0 mAh g^−1^ at 70°C, and achieved high capacity retention of 95.5% after cycling at 10°C for 100 cycles. The polarization of the electrode was inhibited by the introduction of rGO sheets. Meanwhile, compared with the pristine NVP electrode, NVP/G8 electrode exhibited small resistance and high diffusion coefficient of sodium ions.

## Introduction

With the exhaustion of the traditional resources and the increasing environmental problems, researchers expect to develop new clean energy and related storage systems (Xiao et al., 2015; Yang et al., 2016; Luo et al., 2017; Yang Z. H. et al., 2017; Zheng et al., 2017). Lithium ion batteries (LIBs) have being used as the energy storage systems in new energy vehicles. However, the lithium resource cannot meet the requirement of the increasing market. Sodium ion batteries (SIBs) attract the attention of the researchers. Sodium possesses similar chemical properties as lithium. Furthermore, sodium resource is abundant on earth and the price is cheap. Thus, SIBs are considered as the most promising competitive alternative to LIBs (Jian et al., 2014; Wang H. et al., 2015; Chao et al., 2016; Xu et al., 2016). Developing SIBs with excellent electrochemical performance becomes necessary and challenging.

The cathode materials are the key restraining factor of the electrochemical performance in LIBs and SIBs. The electrochemical performances of the batteries, such as energy density, cycle and rate performance, mainly depend on the properties of cathode materials. In SIBs, the diffusion of sodium ions is more difficult compared with that of lithium ions due to the relative larger sodium ion radius (Li et al., 2015). In this case, large volume change during the sodium ion insertion/extraction process leads to structure collapse of the cathode materials, causing poor rate and cycle performance. Various cathode materials, such as Na_0.44_MnO_2_ (Sauvage et al., 2007), Na_4_Fe(CN)_6_ (Qian et al., 2012), Na_2_CoP_2_O_7_ (Barpanda et al., 2013), and NaFeF_3_ (Yamada et al., 2011), had been investigated for SIBs to improve the cycle performance and rate capability (Fang et al., 2015; Wang J. et al., 2015; He et al., 2016; Zhang Y. et al., 2016; Zhang Z. et al., 2016). In the reported cathode materials, Na_3_V_2_(PO_4_)_3_ (NVP) is considered as one of the most promising cathode materials for SIBs (Zhang et al., 2017a; Chen et al., 2018). NVP has a NASICON framework and exhibits high energy density, high operating voltage and excellent thermal stability. Unfortunately, the poor electronic conductivity of the phosphate salt electrode materials also leads to bad rate capacity and cycle performance (Liang et al., 2017). Many methods are applied to improve the electrochemical performance of NVP cathodes, such as cation doping, particle size reduction and conductive materials coating (Qu et al., 2014; Li et al., 2015; Xu et al., 2016; Liang et al., 2017). Among these methods, carbon coating and particle size reduction are the most effective methods. The sodium ion diffusion path can be shortened by diminishing particle size, and the volume change during the charge and discharge process was also reduced (Lin et al., 2013; Qu et al., 2014; Xiao et al., 2014). The electronic conductivity of NVP material can be enhanced effectively by carbon coating (Li et al., 2014). Graphene belongs to carbon conductive materials, which owns superior electronic conductivity and high surface area (Zhang et al., 2014; Li et al., 2016; Yang C. et al., 2017). The electronic conductivity of cathode materials can be obviously enhanced through grapheme coating. Graphene is also used as a template to diminish the particle size (Fang et al., 2016; Xu et al., 2016). Herein, we report a novel NVP microsphere cathode material. Reduced graphene oxide (rGO) decorated NVP (NVP/rGO) microsphere materials were synthesized through spray drying method. In this structure, the NVP microspheres were coated and embedded by rGO sheets. The rGO sheets and amorphous carbon formed carbon conductive network, which improve the electronic conductivity. Consequently, the electrochemical performances of NVP/rGO were improved significantly. To explore the as-prepared NVP/rGO materials, the affecting factors on electrochemical properties, such as morphology, crystal structure and resistance, were systematically investigated.

## Materials and methods

**Figure 1:**
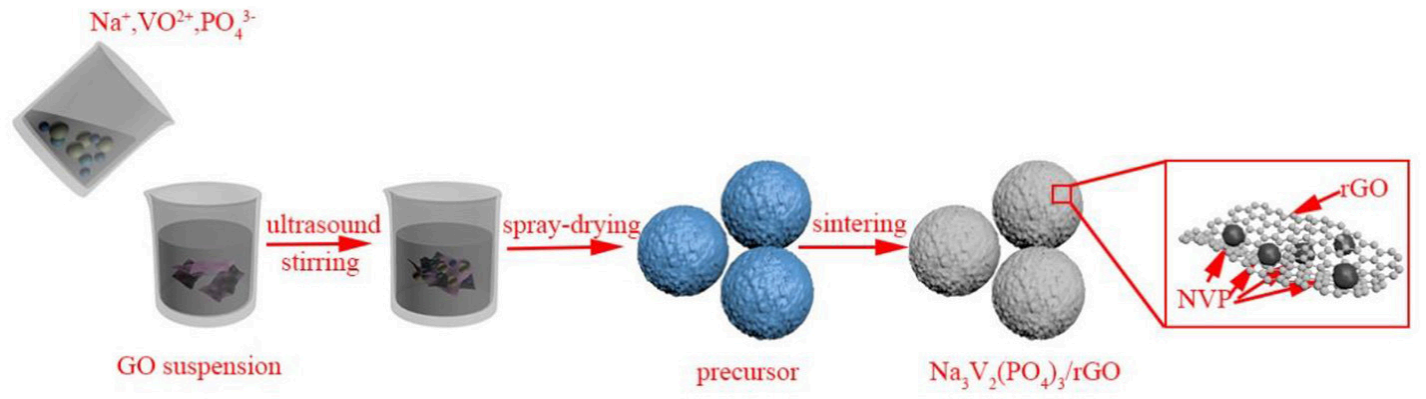
Schematic illustration of synthetic process of NVP/rGO material.

In this study, rGO sheets were prepared by Hummers methods from nature flake graphite. NVP/rGO microspheres were prepared by spray-drying method. Figure 1 illustrates the synthetic process of NVP/rGO microspheres. In a typical experiment, 15 mmol NaHCO_3_, 10 mmol NH_4_VO_3_, 15 mmol NH_4_H_2_PO_4_, and 30 mmol citric acid were dissolved together in the deionized water by stirring. Then, the solution was heated to 80°C and kept for about 30 min. After that, the solution was cooled down to room temperature, and it was mixed with the suspension of graphene oxide (GO) by ultrasound and stirring. The obtained slurry was spray dried at inlet and outlet temperature kept around at 250 and 150°C to get precursor powder. Then, the precursor was calcined at 800°C for almost 10 h in the inert atmosphere. The reduction reaction from GO to rGO occurred simultaneously, and finally the NVP/rGO materials were synthesized. NVP/rGO samples with different content (about 4, 8, and 12 wt%) of rGO was labeled as NVP/G4, NVP/G8, and NVP/G12, respectively. For comparison, NVP without rGO (labeled as NVP/C) was prepared through the same way.

The crystal phases of the prepared materials were studied by X-ray diffraction (XRD, Rigaku D/Max 200PC, Japan) using Cu Kα radiation, with the scanning range of 10–60°. Thermal gravimetric analysis (TGA) of the samples was obtained by a SDT Q600TGDTA apparatus from room temperature to 650°C in air, with the heating rate of 5°C min^−1^. The Raman spectra were investigated by Raman spectroscopy (HORIBA JOBIN YVON, France). The chemical valence states of the carbon were studied by X-ray photoelectron spectroscopy (XPS, ESCALAB 250Xi, Thermo Fisher Scientific Co., Ltd, USA). The sample morphologies were observed by scanning electron microscopy (SEM, Nova NanoSEM-230, USA), and the microstructures were observed by high-resolution transmission electron microscopy (HRTEM, FEI Tecnai G2 F20 S-Twin, USA), working at 200 kV. The prepared NVP samples were evaluated using coin cells fabricated in a glovebox, with sodium foil as the counter electrode and a glass microfiber (Whatman GF/D) as the separator. The sample powder, carbon black and polyvinylidene fluoride (mass ratio 8:1:1) were mixed in N-methylpyrrolidinone to get the slurry. The aluminum foil pasted with the slurry was dried in the oven, 120°C for 4 h. The electrolyte was 1 M NaClO_4_ dissolved in ethylene carbon and dimethyl carbonate (1:1), and 5 vol% fluoroethylene carbonate. Electrochemical measurements were performed by a constant current/constant voltage method, with the potential of 2.0–3.8 V (vs. Na^+^/Na). Electrochemical impedance spectroscopy (EIS) tests were carried out with an electrochemical workstation (CHI 660D, CH Instruments, China), with the frequency range of 10^−2^-10^5^ Hz. Cyclic voltammetry (CV) measurements were also studied with this workstation, and the scan rate of which was 0.1 mV s^−1^.

## Results and discussion

**Figure 2:**
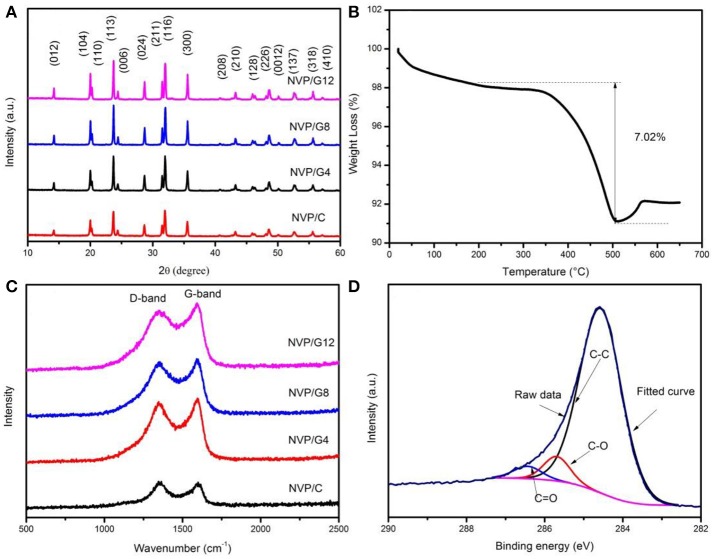
**(A)** XRD patterns of NVP/rGO samples; **(B)** TGA curve of NVP/G8 sample; **(C)** Raman spectrum of NVP/rGO samples; **(D)** C1s XPS analysis spectrum of NVP/G8 sample.

NVP/rGO samples were prepared by spray-drying method. To investigate the effect of rGO sheets on the structure, the microspheres were characterized by XRD. The XRD patterns of NVP/rGO samples were shown in Figure 2A. All diffraction peaks are indexed to the R3c space group of the rhombohedral NASICON structure without any impurities. It indicates that the crystal structure of NVP was not changed by rGO embedding. The carbon contents of NVP/rGO samples were measured with carbon-sulfur analyzer, which were 1.00 (NVP/C), 4.53 (NVP/G4), 7.11 (NVP/G8), and 10.11 (NVP/G12) wt%, respectively. The carbon of NVP/C derived from the decomposition of citric acid (Chen et al., 2018). The content of the NVP/G8 was also confirmed by TGA test (Figure 2B), and carbon content was 7.02 wt%, which is close to the result of carbon-sulfur analyzer.

Raman spectroscopy was used to investigate the characteristics of the carbon materials. Figure 2C displays the Raman spectra of NVP/rGO samples. The D (disorder-induced phonon mode) and G (graphite band) bands were observed around 1,300 and 1,500 cm^−1^ for all samples, respectively. The relative intensity ratio (I_D_/I_G_) indicates the degree of structural disorder of the carbon materials. The I_D_/I_G_ ratio values of NVP/C, NVP/G4, NVP/G8, and NVP/G12 samples were 1.02, 0.95, 0.93, and 0.90, respectively. It is found that the I_D_/I_G_ ratio values decreased with the increase of rGO sheet content in the samples, and the I_D_/I_G_ ratio value of NVP/G8 material was the lowest. The lower I_D_/I_G_ ratio value suggests the graphitization degree of the material is higher, and the electric conductivity of the electrode materials is also higher (Xu et al., 2016). Figure 2D shows the C1s XPS analysis spectrum of NVP/G8 material. The fitted curve was resolved into three peaks (blue, red, and black), and the binding energies of 284.5, 285.7, and 286.5 ev corresponds to C-C, C-O, and C = O functional groups, respectively (Fang et al., 2016).

**Figure 3:**
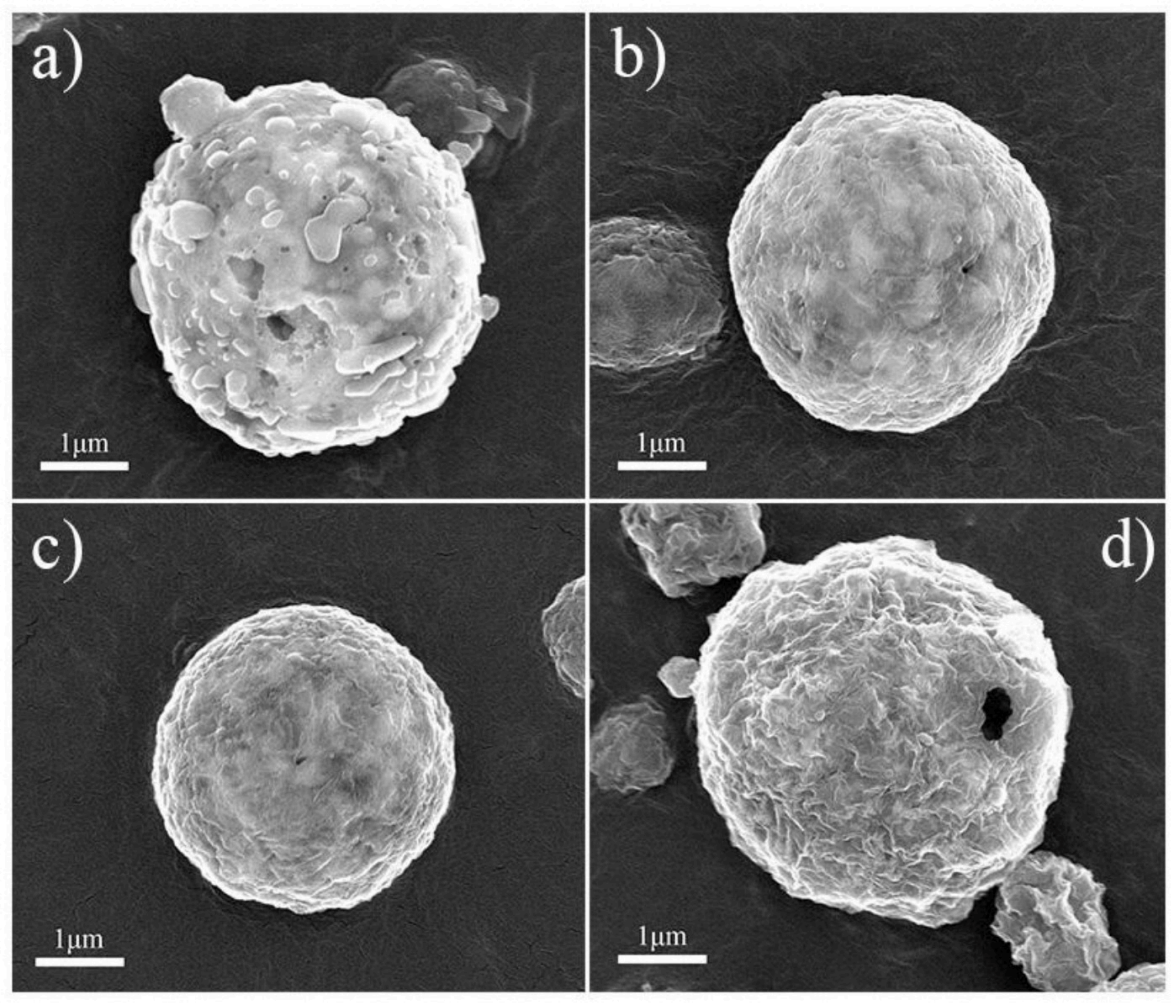
SEM images of **(a)** NVP/C, **(b)** NVP/G4, **(c)** NVP/G8, and **(d)** NVP/G12 samples.

**Figure 4:**
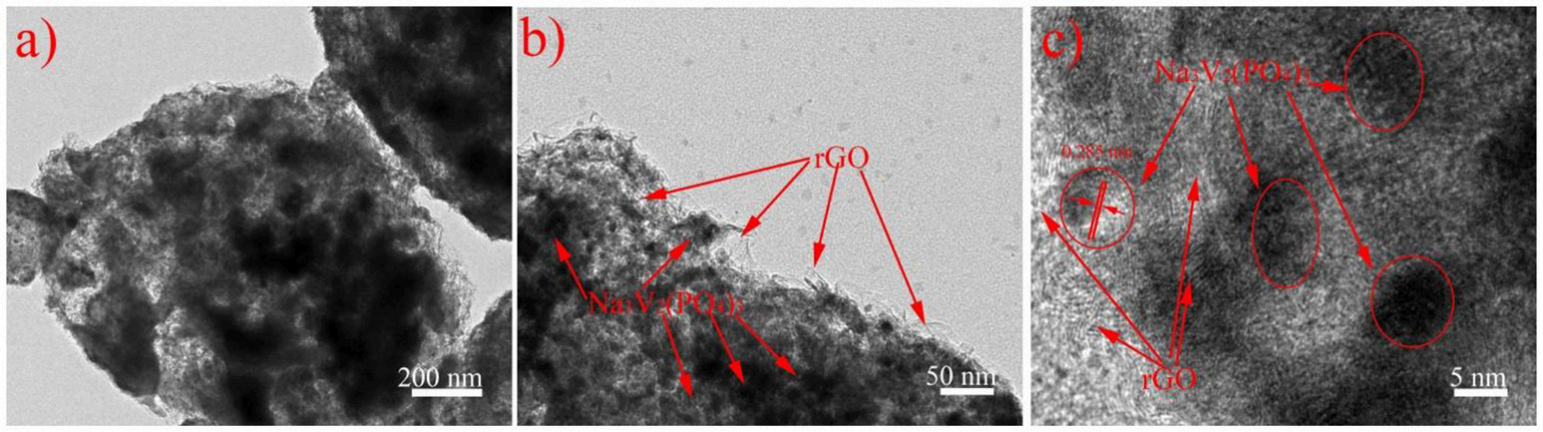
**(a,b)** TEM and **(c)** HRTEM images of NVP/G8 sample.

**Figure 5:**
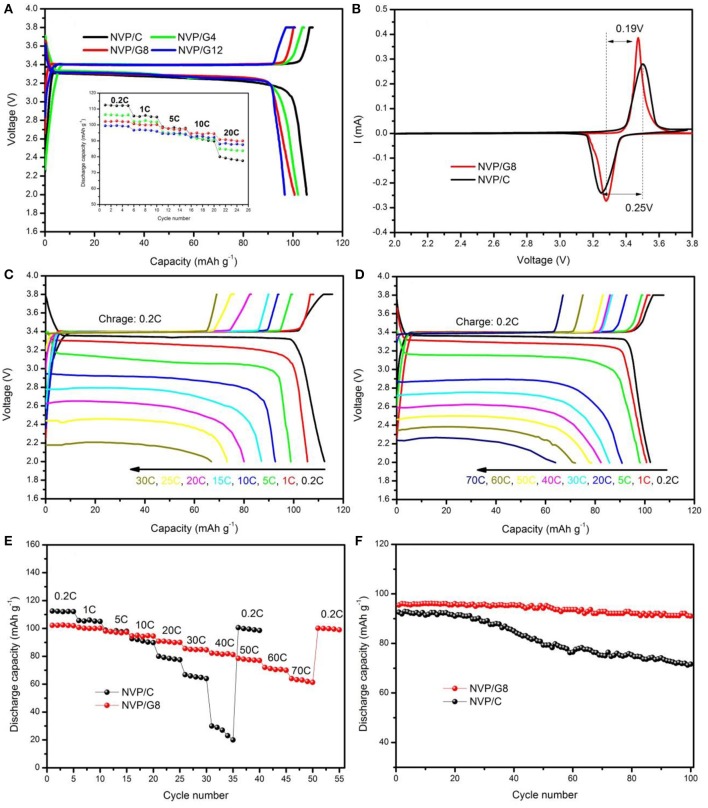
**(A)** Charge and discharge curves of NVP/rGO electrodes; the inset is the rate performance of the electrodes; **(B)** CV curves of NVP/C and NVP/G8 electrodes; charge and discharge curves of **(C)** NVP/C and **(D)** NVP/G8 electrodes at various current rates; **(E)** rate performance of NVP/C and NVP/G8 electrodes at various current rates; **(F)** cycle performance of NVP/C and NVP/G8 electrodes at 10°C.

Figure 3 shows the SEM images of NVP/rGO materials. It is seen that all the NVP/rGO particles of the samples were spherical, and the size distribution was from 0.2 to 1 μm. It is found that there are some secondary particles on the surface of the microsphere in Figure 3a, however, the secondary particles are hardly seen in Figures 3b–d, as the rough surface of the microspheres were covered with rGO sheets. The inner structure of NVP/rGO microspheres was investigated by TEM and HRTEM. Figures 4a,b shows the low magnification TEM image of NVP/G8 microsphere. It is found that the surface of the small particles was covered with rGO sheets, and which were also embedded in the microsphere. The secondary particle size of NVP/G8 is smaller than 100 nm, as the microsphere was separated by rGO sheets and the particle growth was controlled. The HRTEM image (Figure 4c) shows that the crystal lattice spacing of NVP was about 0.285 nm, corresponding to (211) plane of NVP. The functional groups of GO, such as -COOH and -OH, reacted with Na^+^ and ${\text{PO}}_{4}^{3-}$ to make the electron transport easily. In the TEM images, it is further confirmed that rGO sheets distributed uniformly in the microspheres. The carbon conductive network consisted of rGO sheets and amorphous carbon creates good interfacial contact among NVP particles, which is benefit for accelerating the electron transport in the electrode material. The electrochemical performances of all the samples were also investigated, as shown in Figure 5. Figure 5A shows the charge and discharge curves of NVP electrodes with different content of rGO sheets, the charge and discharge rates were 0.2 and 1 C (1 C = 110 mA g^−1^), respectively. The discharge capacity of NVP/C, NVP/G4, NVP/G8, and NVP/G12 were 105.6, 102.1, 100.7, and 96.7 mAh g^−1^, respectively. The discharge capacity decreased with the increase of the rGO content, as rGO did not involve the electrochemical reaction. The inset figure in Figure 5A shows the rate performances of all the samples. All the NVP/rGO samples showed better rate performances than NVP/C sample. And, the NVP/G8 sample had the best rate performance. It suggests that the rate performance is enhanced due to the introduction of rGO sheets. For investigating the effect of rGO sheets on intercalation/deintercalation behavior of sodium ions, the NVP/C and NVP/G8 electrodes were evaluated by CV, as displayed in Figure 5B. The CV curves of the samples were similar. The redox couple located at about 3.15 and 3.50 V, which are attributed to the V^4+^/V^3+^ redox couple(Liang et al., 2017). The potential differences of the redox peaks of NVP/C and NVP/G8 electrodes were 0.25 and 0.19 V, respectively. The lower difference between the oxidation and the reduction peaks suggests the smaller degree of the electrode polarization. Furthermore, the redox peaks of NVP/G8 electrode became sharper. It indicates that the introduction of rGO sheets can decrease the polarization. The galvanostatic charge and discharge process of the NVP/C and NVP/G8 electrodes was studied at different rates and shown in Figures 5C,D. It is seen that the NVP/G8 electrode delivered capacities of 102.2, 100.7, 98.1, 90.8, 85.8, 82.3, 78.5, and 72.0 mAh g^−1^ at 0.2, 1, 5, 20, 30, 40, 50, and 60°C, respectively; even at 70°C, the electrode still had discharge capacity of 64 mAh g^−1^. As a contrast, NVP/C electrode delivered capacities of 112.3, 105.6, 98.8, 92.6, 87.0, 80.0, 73.1, and 66.8 mAh g^−1^ at 0.2, 1, 5, 10, 15, 20, 25, and 30°C, respectively. Although NVP/C electrode delivered high capacity at low rate, it did not work at high rate (above 30°C). It is concluded that the potential polarization of NVP/G8 is smaller than the NVP/C, resulting in the improvement of discharge capacities. Figure 5E shows the rate properties of NVP/C and NVP/G8 electrodes. The initial discharge capacity of NVP/G8 was lower than that of NVP/C at low rate, as rGO has no contribution to the capacity. When the current density was higher than 5°C, NVP/G8 electrode showed higher discharge capacity than NVP/C electrode. With the increase in discharge current density, the capacity of NVP/C decreased quickly. In contrast, the capacity of NVP/G8 decreased slowly. Even the rate up to 70°C, the capacity retention still kept at 62.6% (relative to the capacity at 0.2°C). When the rate decreased to 0.2 C, the discharge capacity of NVP/G8 electrode almost increased to the value of initial capacity. The improvement of rate performance implies that carbon network can enhance the electronic conductivity of the NVP materials. To further investigate the electrochemical performance of NVP samples, the cycle performances of NVP/C and NVP/G8 were evaluated at 10°C (Figure 5F). The initial discharge capacities of NVP/C and NVP/G8 at 10°C were 92 and 95.5 mAh g^−1^, respectively; after 100 cycles, which decreased to 71.6 and 91.0 mAh g^−1^, with the capacity retentions of 77.8 and 95.5%, respectively. It is demonstrated that the cycle performance was obviously improved by the addition of rGO sheets.

**Figure 6:**
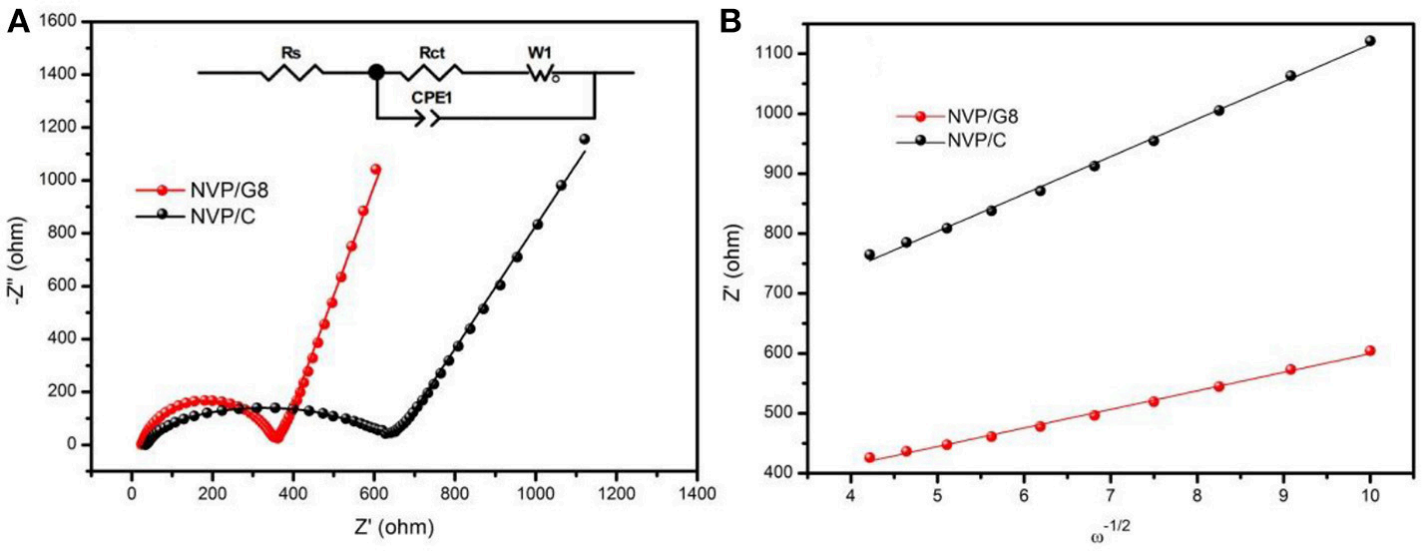
**(A)** Nyquist plots of NVP/C and NVP/G8 electrodes; the inset is the equivalent circuit for EIS result fitting; **(B)** the relationship between Z′ and ω^−1/2^ in the low-frequency region.

For understanding the effect of rGO introduction on electrode reaction kinetics, the NVP/C and NVP/G8 electrodes were investigated by EIS. Figure 6A illustrates Nyquist plots of the two electrodes. Each plot consists of a semicircle and a straight line at high-middle and low frequency area, respectively. The intercept at high frequency area corresponds to the ohmic resistance of the electrode and electrolyte. The semicircle corresponds to charge-transfer resistance, while the straight line related to sodium ion diffusion in the NVP particles. The equivalent circuit model is shown in the inset figure of Figure 6A. In this model, R_s_ corresponds to the ohmic resistance, R_ct_ corresponds to the charge-transfer resistance, CPE and W represents the double-layer capacitance and Warburg impedance with regard to sodium ion diffusion in the NVP particles. The impedance parameters are listed in Table 1. It is found that R_s_ and R_ct_ of NVP/G8 electrode were much smaller than those of NVP/C electrode. It suggests that the charge-transfer speed through the electrode and electrolyte interface is increased by rGO introduction. Furthermore, the diffusion coefficient of sodium ions (D_Na+_) was calculated by the equations as follows (Zhang et al., 2015a,b, 2017b):

(1) $$\begin{array}{rcl} {\text{D}}_{\text{N}{\text{a}}^{+}} & = & \frac{{\text{R}}^{2}{\text{T}}^{2}}{2{\text{A}}^{2}{\text{n}}^{4}{\text{F}}^{4}{\text{C}}^{2}\sigma^{2}} \end{array}$$

(2) $$\begin{array}{rcl} {\text{Z}}_{\text{real}} & = & {\text{R}}_{\text{s}}+{\text{R}}_{\text{ct}}+\sigma \omega^{-1/2} \end{array}$$

where R represents the gas constant, T is the absolute temperature, A is the surface area of the cathode, n signifies the number of transferred electrons, F is the Faraday constant, C is the concentration of sodium ions, σ is the Warburg coefficient, and ω is the angular frequency in the low frequency region. The linear fitting of *Z*′ versus ω^−1/2^ is shown in Figure 6B, in which the value of σ is the slope. As presented in Table 1, D_Na+_ of the NVP/G8 and NVP/C electrode was 1.24 × 10^−13^ and 8.72 × 10^−14^ cm^2^ s^−1^, respectively. The NVP/G8 electrode showed higher value of sodium ion diffusion coefficient, as the sodium ion diffusion path was reduced in the small particles and the contact area between NVP and the electrolyte increased. The R_s_ and R_ct_ of NVP/G8 electrode both became smaller, as the electron conductivity was enhanced by rGO sheets. Therefore, lower R_s_ and R_ct_, and higher D_Na+_ are benefit for improving the electrochemical performance.

**Table 1:** Impedance parameters of NVP/C and NVP/G8 electrodes obtained from equivalent circuit fitting.

**Samples**	**R_s_(Ω)**	**R_ct_(Ω)**	**σ(Ω)**	**D_Na+_(cm^2^ s^−1^)**
NVP/C	33.8	715.8	62.4	8.72 × 10^−14^
NVP/G8	24.0	399.0	31.0	1.24 × 10^−13^

## Conclusion

In summary, NVP microspheres decorated with different content of rGO sheets were synthesized by spray-drying method. GO plays as a binder during the spray-drying process. The carbon network creates good interfacial contacts among NVP particles and accelerates the electron transport. By introduction of rGO sheets into NVP microspheres, the electrochemical properties of NVP/rGO materials were all obviously enhanced, especially at high rates. NVP/G8 electrode exhibited the capacities of 102.2 mAh g^−1^ at 0.2°C, and 64 mAh g^−1^ at 70°C. Even cycled at 10°C for 100 cycles, the capacity retention of NVP/G8 electrode remained 95.5%. The significant improvement in electrochemical performance of NVP sample is attributed to the lower resistance and higher D_Na+_ compared with those of the pristine sample. Consequently, NVP microspheres decorated with rGO sheets may be a promising cathode material to achieve superior electrochemical performance for energy storage.

## Author contributions

HC carried out the experiment and wrote the manuscript; YH and GM participated in the material preparation; HT supervised all the experiments and proofread the manuscript; WY, JZ, and ZD contributed to the discussion.

### Conflict of interest statement

The authors declare that the research was conducted in the absence of any commercial or financial relationships that could be construed as a potential conflict of interest.
